# Similarities and Differences of Photosynthesis Establishment Related mRNAs and Novel lncRNAs in Early Seedlings (Coleoptile/Cotyledon vs. True Leaf) of Rice and *Arabidopsis*

**DOI:** 10.3389/fgene.2020.565006

**Published:** 2020-09-08

**Authors:** Yafei Shi, Jian Chen, Xin Hou

**Affiliations:** State Key Laboratory of Hybrid Rice, College of Life Sciences, Wuhan University, Wuhan, China

**Keywords:** rice, *Arabidopsis*, messenger RNA, long non-coding RNA, cotyledon, true leaf, photosynthesis

## Abstract

Photosynthesis uses sunlight and carbon dioxide to produce biomass that is vital to all life on earth. In seed plants, leaf is the main organ for photosynthesis and production of organic nutrients. The seeds are mobilized to fuel post-germination seedling growth until seedling photosynthesis can be efficiently established. However, the photosynthesis and metabolism in the early growth and development have not been studied systematically and are still largely unknown. In this study, we used two model plants, rice (*Oryza sativa* L.; monocotyledonous) and *Arabidopsis* (*Arabidopsis thaliana*; dicotyledonous) to determine the similarities and differences in photosynthesis in cotyledons and true leaves during the early developmental stages. The photosynthesis-related genes and proteins, and chloroplast functions were determined through RNA-seq, real-time PCR, western blotting and chlorophyll fluorescence analysis. We found that in rice, the photosynthesis established gradually from coleoptile (cpt), incomplete leaf (icl) to first complete leaf (fcl); whereas, in *Arabidopsis*, photosynthesis well-developed in cotyledon, and the photosynthesis-related genes and proteins expressed comparably in cotyledon (cot), first true leaf (ftl) and second true leaf (stl). Additionally, we attempted to establish an mRNA-lncRNA signature to explore the similarities and differences in photosynthesis establishment between the two species, and found that DEGs, including encoding mRNAs and novel lncRNAs, related to photosynthesis in three stages have considerable differences between rice and *Arabidopsis*. Further GO and KEGG analysis systematically revealed the similarities and differences of expression styles of photosystem subunits and assembly factors, and starch and sucrose metabolisms between cotyledons and true leaves in the two species. Our results help to elucidate the gene functions of mRNA-lncRNA signatures.

## Introduction

Seeds provide energy and materials for the germination, early growth and development of plants. The size and storage of seeds vary considerably among species. In monocots, the endosperm constitutes the majority of the mature seed (Berger et al., 2006). Rice (*Oryza sativa*) endosperm consists of an outer aleurone layer and an inner starchy endosperm (Becraft and Asuncion-Crabb, 2000; Krishnan and Dayanandan, 2003). In maize (*Zea mays*), the endosperm typically consists of the vitreous endosperm and the starchy endosperm (Wang et al., 2012). In dicotyledons, such as the oilseed crop *Brassica napus* (rapeseed) and the model plant *Arabidopsis*, storage products consist of proteins and triacylglycerols (TAGs) synthesized mainly in the embryo (Baud et al., 2008). The endosperm and embryo are mobilized to fuel post-germination seedling growth until seedling photosynthesis can be efficiently established (Sela et al., 2020).

Rice, as a monocotyledonous model plant, is one of the most important crops in the world. Rice grain, as a cereal crop, is a food reserve to nourish growing seedlings during germination (Zheng and Wang, 2015; Wu et al., 2016). The starchy endosperm is the major energy source of rice and contains mostly starch and a small amount (8%) of proteins (Becraft and Yi, 2011). During rice seed germination, starch is decomposed into glucose, which is directly used as raw material for respiration, and cellulose is further synthesized to ensure the formation of cell walls in new cells (Leonova et al., 2010). Proteins stored in seeds are decomposed into soluble amino acids under the action of protease and transported to the growth site of embryos. Amino acids under the action of corresponding enzymes form structural proteins, which become cellular components in buds and roots. Rice seeds contain less fat, that is, approximately 1–2%. During seed germination, storage lipids are decomposed into fatty acids and glycerol by lipase and then converted into sugars, thereby providing raw materials for respiration. *Arabidopsis* seeds are considerably smaller than rice. Rice (Nipponbare) seed usually weighs 20 mg. By contrast, *Arabidopsis* (Col-0) seed weighs approximately 20 μg. *Arabidopsis* seeds contain 30–40% proteins (Baud et al., 2002), 38% model oil (O’Neill et al., 2003), and 2% oligosaccharides (Bentsink et al., 2000). The seed stored oil, TAGs, is metabolized to provide carbon skeleton and energy resources for germination and growth of young seedlings (Baud et al., 2002). At the stage of seeds maturation, the most abundant mRNAs in embryos are those encoding seed storage proteins, which are considered to be the source of seedling nitrogen for young seedlings (Pang et al., 1988).

The photosynthesis and carbon cycle of plants are the basis of all life activities. Photosynthesis plays a crucial role in biology and biogeochemistry, transforming sunlight energy and CO_2_ into metabolic products of life (Liu and Last, 2017). After germination, leaves are the main organs of plant photosynthesis. In dicotyledonous plants, such as *Arabidopsis*, cotyledons are components of seeds and the first pair of leaves; the first true leaves appeared several days later. Both cotyledons and true leaves contain chloroplasts and have photosynthetic activity; however, the differentiation of chloroplasts follows different pathways in these two organs (Deng and Gruissem, 1987; Mansfield and Briarty, 1996). Cotyledons are capable of storing nutrients and performing photosynthesis and provide a major proportion of matter needed for seedling growth and development until the first true leaf becomes a significant exporter of photosynthates (Zhang et al., 2008; Zheng et al., 2011). Cotyledon deficiency experiments result in growth arrest or even death of young seedlings (Garcia-Cebrian et al., 2003; Hanley and May, 2006). The cotyledon chloroplast biogenesis factor CYO1 (shi-yo-u means cotyledon in Japanese) associated with PSI/LHCI and PSII/LHCII complexes is crucial for the biogenesis of cotyledon chloroplasts in *Arabidopsis* (Shimada et al., 2007; Muranaka et al., 2012). In rice, the coleoptile is the first leaf-like organ formed around the embryo during embryogenesis (Hirano, 2008). It is the basic structure of the seedling and plays a protective role when the embryo passes through the soil layers (Xiong et al., 2017). The incomplete leaf is the first green organ with only a sheath but no blade. The complete leaves with blades and sheaths are from the second green leaf (Du et al., 2018). OsCYO1 is expressed in leaf but not in coleoptile, and functions in the accumulation and/or assembly of PSI (Tominaga et al., 2016). There are structural and functional differences between monocotyledonous and dicotyledonous plants in early growth and development. In rice, photosynthetic activity may differ from that in *Arabidopsis*. However, the similarities and differences in photosynthesis and metabolism in the early growth and development of the two model plants have not been studied systematically and are still largely unknown.

In recent years, deep sequencing of transcripts has been increasingly used in the identification of differential expression (Lu et al., 2010; Bhargava et al., 2013; Gao et al., 2013; Yuan et al., 2016). Long non-coding RNAs (lncRNAs) are RNAs with lengths greater than 200 bp that lack protein-encoding functions and play important roles in essential biological processes. lncRNA mostly functions in the nucleus, often in association with chromatin processes (Chekanova, 2015). lncRNAs can be divided into three categories: long intergenic ncRNAs (lincRNAs), intronic ncRNAs (incRNAs), and natural antisense transcripts (NATs) transcribed from the complementary DNA strand of the associated genes (Chekanova, 2015). Although lncRNAs do not have the ability to encode proteins, it is widely believed that lncRNAs can be transcribed and regulate gene expression at the transcriptional and post-transcriptional levels (Kim and Sung, 2012). Many plant lncRNAs are developmentally and environmentally regulated and likely represent functional components of the transcriptome (Liu et al., 2012). The regulation of coding genes mediated by lncRNA at the transcriptional level can be divided into *cis*-regulation and trans-regulation. Epigenetic regulation of lncRNAs plays a key role in flowering regulation by controlling the expression of FLC (flowering site C) in *Arabidopsis*. FLC plays a key role in regulating flowering process and regulates flowering independently of environmental signals (Amasino and Michaels, 2010). COLDAIR and COOLAIR are two different classes of lncRNAs transcribed from FLC that participate in epigenetic silencing of FLC (Liu et al., 2010; Heo and Sung, 2011; Tian et al., 2019). A lncRNA HID1 with a length of 236 nt length was identified by chain-specific transcriptome sequencing. This lncRNA participates in seed photomorphogenesis by mediating PIF3 expression in *Arabidopsis* (Wang et al., 2014b). In addition, some studies have found that lncRNAs can be transcribed from the antisense strands of genes. These lncRNAs are usually involved in post-translational splicing, editing, transport, translation, and degradation of mRNA (Liu et al., 2019).

In this study, high-throughput sequencing was used to analyze mRNA, novel lncRNAs and their target genes during the early growth and development of rice and *Arabidopsis*. We show that photosynthetic protein complexes gradually mature from cpt, icl to fcl in rice. However, the functions are fully developed during the cot stage of *Arabidopsis*. Photosynthesis and photosynthesis-related processes were clustered in the three stages of rice but not in *Arabidopsis* through Gene Ontology (GO) and Kyoto Encyclopedia of Genes and Genomes (KEGG) analyses of differentially expressed genes (DEGs). Further analysis of DEGs, including mRNAs and lncRNAs, showed that the upregulated genes from icl to fcl were distributed in photosystem II (PSII), photosystem I (PSI), cytochrome b6/f complex, photosynthetic electron transport and F-type ATPase in rice but not in *Arabidopsis*. Additionally, assembling the individual subunits to form PSII and PSI are also different in the two plants and may not function at the transcriptional level.

## Materials and Methods

### Plant Materials and Growth Conditions

Rice (Nipponbare) were grown under 16 h of light at 30°C and 8 h of darkness at 28°C. Seedlings were grown on plates containing only water and 0.8% agar. Leaves were harvested at 68 h for coleoptile (cpt), 82 h for incomplete leaf (icl), and 127 h for first complete leaf (fcl). *Arabidopsis* (Columbia-0) was grown under 16 h of light at 22°C and 8 h of darkness at 20°C. Seedlings were grown on 1/2 MS medium containing 1.0% sucrose (pH 5.7) or only water and 0.8% agar. Leaves were harvested at 111 h for cotyledon (cot), 183 h for first true leaf (ftl), and 236 h for second true leaf (stl).

### RNA Extraction and RNA-Seq Preparation

Approximately 60 rice seedlings and 600 *Arabidopsis* seedlings were collected from each stages and frozen in liquid nitrogen immediately. Three biological replicates were performed. Total RNA was extracted using a RNeasy Plant Mini Kit followed by DNase I treatment according to the manufacturer’s manual (Qiagen). The extracted RNA was analyzed by 1% (w/v) agarose gel for quality and completeness, and the concentration was measured on a NanoDrop 2000c spectrophotometer (NanoDrop Technologies, Wilmington, DE, United States). The RNA samples were sent to Novogene (Novogene, Co. Ltd.) for mRNA purification, cDNA library construction and sequencing using the platform Illumina. The original raw data from HiSeq X Ten was transformed to sequenced reads by base calling. Sequencing data analysis was completed in UniqueGene (Wuhan, China).

### Sequence Analysis

Reads qualities of the RNA sequencing (RNA-Seq) were evaluated using FastQC (Version 0.11.5). The clean data was evaluated by removing the adapter and low-quality reads of the raw data obtained from Illumina sequencing. The clean reads were searched against the rice and *Arabidopsis* genome Using HISAT2 (Version 2.0.4). The obtained transcripts were assembled using StringTie (Version 1.0.4). All downstream analyses were based on the high quality clean data.

### Identification of Novel lncRNAs

Based on the structural characteristics and non-coding function of lncRNA, we established a five-step screening method to obtain high quality lncRNA: (1) Transcripts with one exon were removed while the transcripts with two or more exons were selected; (2) the transcripts with length < 200 bp were removed; (3) Filtered out transcripts with reads coverage < 5 in all samples; (4) Filtered out the mRNA and other non-coding RNA (rRNA, tRNA, snoRNA, snRNA, etc.) by comparing gffcompare (Version 0.10.4) with the annotation files; (5) The potential lincRNA, intronic lncRNA and anti-sense lncRNA were screened according to the class_code information (”u,” “i,” “x”) in the comparison results. Based on the length of the open reading frame, homology with known proteins and their coding potential, the CNCI (Coding-Non-Coding Index) (Sun et al., 2013), CPC (Coding Potential Calculator) (Kong et al., 2007), and CPAT (Coding Potential Assessment Tool), which rapidly recognizes coding and non-coding transcripts from a large pool of candidates (Wang et al., 2013) were combined to screen the lncRNAs. This analysis was combined with information from the Pfam protein database to ensure the predicted lncRNA transcripts did not contain protein-coding domains (Mistry et al., 2007). The transcripts that meet these four criteria were determined to be lncRNAs.

### Differential Expression Analysis of mRNAs and lncRNAs

The clean reading was aligned with the reference genomes by HiSAT2 software, and then gene and transcript expression levels were calculated by performing RSEM. In order to make the data of gene expression comparable among samples, the level of gene expression was standardized. FPKM (fragments per kilobase of exon per million fragments mapped) values were used to analyze gene expression. Differential expression analysis of different stages was performed using the DESeq R package (1.10.1). DESeq provides a statistical program based on negative binomial distribution to determine differential expression in digital gene expression data. The resulting *P-*values were adjusted by Benjamini and Hochberg’s approach for controlling the false discovery rate (Benjamini and Hochberg, 1995). Genes with an adjusted *P*-value < 0.05 and Fold change ≥ 2.00 found by DESeq were assigned as differentially expressed.

### Target Genes of Differentially Expressed lncRNAs

The differentially expressed lncRNAs were selected as targets by *cis*- or *trans*- regulation. In the prediction of *cis* pathway target genes, the genes transcribed in 10 kb upstream or downstream of coding genes. Trans-acting target genes were selected by RNAplex software (Tafer and Hofacker, 2008).

### GO and KEGG Pathway Analyses of Differentially Expressed mRNAs and lncRNAs

The GO enrichment analysis of DEGs was implemented by Wallenius non-central hypergeometric distribution based on GOseq R package (Young et al., 2010), which can be adjusted according to the gene length bias in DEGs. GO terms *P*-value < 0.05 were considered significantly enriched by differentially expressed genes. KEGG (Kanehisa and Goto, 2000) is a database resource for understanding the high-level functions and utilities of biological systems, such as cells, organisms and ecosystems, from molecular-level information, especially large-scale molecular datasets generated by genome sequencing and other high-throughput experimental technologies^1^. The statistical enrichment of DEGs in KEGG pathway were tested using KOBAS (Mao et al., 2005) software. The significantly enriched KEGG pathways were determined using the corrected *P*-value < 0.05 as a threshold.

### Expression Analysis by Quantitative Real-Time PCR (q-RT PCR) of DEGs

The DEG results were confirmed by q-RT PCR using the same RNA samples that were used for RNA library construction. The isolated RNA was reverse transcribed to cDNA using a PrimeScript RT reagent Kit (Takara, Dalian China) according to the manufacturer’s instructions. Real-time PCR analyses were performed on an Applied Biosystems 7300 Plus Real-Time PCR System with SYBR Green Mix (Takara, Dalian, China). The amplified PCR products were quantified and normalized using *actin1* for rice and *actin2* for *Arabidopsis* as a control (Supplementary Table S1). The correlation between RNA Seq and q-RT PCR results were analyzed through pearson’s correlation coefficient (r).

### Immunoblot Analysis

Protein was extracted from each tissue using extraction buffer [50 mM Tris-MES pH 7.5, 50 mM NaCl, 0.5% SDS (v/v)], and protease inhibitor cocktail P9599 (Sigma-Aldrich, St. Louise, MO, United States). The extract was centrifuged at 12000 rpm at 4°C for 30 min. For immunoblotting, protein samples (18 μg total protein) were separated on 12% SDS PAGE gels and transferred to nitrocellulose membranes (BioTrace^TM^ NT nitrocellulose, Mexico) followed by western blot analysis. After blocking non-specific binding with 5% milk, the blot was subsequently incubated with antibodies generated against the indicated proteins and detected using the Super Signal^TM^ West Pico PLUS Chemiluminescent Substrate kit (Thermo Scientific, United States).

### Chlorophyll Fluorescence Measurements

Chlorophyll fluorescence was measured using FluorCam 800 MF (Photon Systems Instruments, spol. s r.o.). Plants were maintained in the dark for 30 min before the measurements. The intensities of the actinic light and saturating light were 300 and 2,100 μmol m^–2^ s^–1^ photosynthetic photon flux density for rice, and 80 and 1,600 μmol m^–2^ s^–1^ photosynthetic photon flux density for *Arabidopsis*, respectively. Three biological replicates for each treatment were performed with 10 plants.

### Statistical Analysis

All data were expressed as mean ± SD and analyzed using GraphPad Prism software (version 7.0, La Jolla, CA, United States). Student’s *t*-test was used to compare the different groups. And the significant differences between the three stages were calculated using Tukey’s test. The Pearson’s correlation coefficient (r) was used to analyze the correlation between qRT-PCR and RNA-seq results. Differences with |log_2_ ratio| ≥ 1 and *P* < 0.05 were selected for the DEGs. Cytoscape software was used to visualize the inferred gene networks (Shannon et al., 2003). Modules were visualized using Cytoscape version 3.6.1.

### Accession Numbers

The sequence data generated in the current study has been uploaded to the NCBI Sequence Read Archive. The accession numbers are SRR10907568–SRR10907573 (coleoptile sequencing of rice), SRR10913120-SRR10913123 (incomplete leaf sequencing of rice), SRR10913307-SRR10913312 (first complete leaf sequencing of rice), SRR10914245-SRR10914250 (cotyledon sequencing of *Arabidopsis*), SRR10914757-SRR10914762 (first true leaf sequencing of *Arabidopsis*), SRR10915176-SRR10915181 (second true leaf sequencing of *Arabidopsis*)^2^.

## Results

### Photosynthesis and Chloroplast Development of Early Growth and Development in Rice and *Arabidopsis*

**FIGURE 1:**
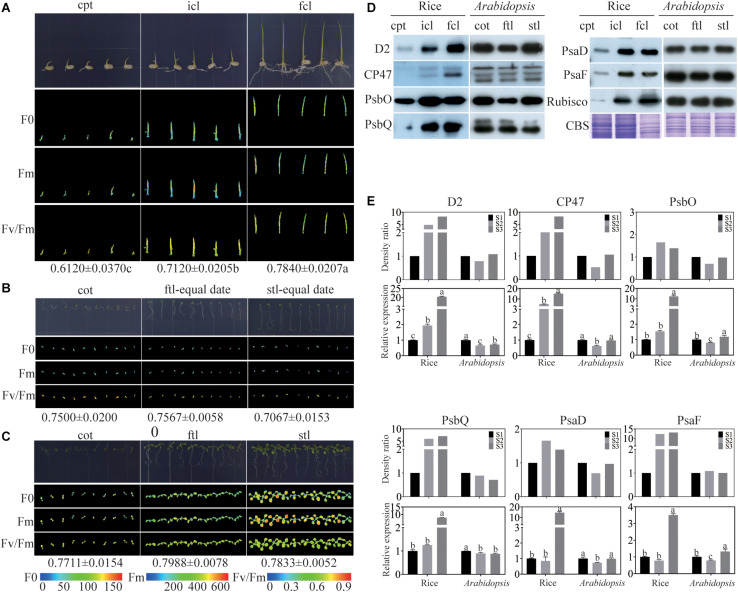
Comparison of rice and *Arabidopsis* at the early developmental stages. **(A)** rice grown on 0.8% agar under 16 h of light at 30°C and 8 h of darkness at 28°C. **(B)** *Arabidopsis* plants grown on 0.8% agar under 16 h of light at 22°C and 8 h of darkness at 20°C conditions for different stages **(C)** *Arabidopsis* plants grown on 1/2MS under 16 h of light at 22°C and 8 h of darkness at 20°C at different stages. **(A–C)** Chlorophyll fluorescence analysis. F_0_, the minimum fluorescence; Fm, the maximum fluorescence; Fv/Fm, the maximum efficiency of PSII photochemistry. cpt, rice coleoptile; icl, rice incomplete leaf; fcl, rice first complete leaf; cot, *Arabidopsis* cotyledons; ftl, *Arabidopsis* first true leaf; stl, *Arabidopsis* second true leaf. **(D)** Analysis of thylakoid membrane proteins in rice and *Arabidopsis* at different stages. 16 μg total proteins were separated by 12% SDS PAGE. And immunoblots were performed using antibodies as indicated. CBS, Coomassie Blue Staining. **(E)** The relative expression levels of chloroplast proteins in **(D)** and the relative expression levels of relavent genes of rice and *Arabidopsis*. Data was presented as the means ± SD of three biological replicates. S1, cpt and cot; S2, icl and ftl; S3, fcl and stl. a, b, and c indicate significant differences between the three stages according to Tukey’s test.

To find the similarities and differences in photosynthesis and chloroplast development between monocotyledons and dicotyledons during early growth and development, photosynthesis was determined by measuring chlorophyll fluorescence in rice and *Arabidopsis*. We collected coleoptile (cpt) before the incomplete leaf (icl) appeared in rice and cotyledon (cot) before the first true leaf (ftl) appeared in *Arabidopsis* as stage 1 (S1), icl before the first complete leaf (fcl) appeared in rice and ftl before the second true leaf (stl) appeared in *Arabidopsis* as stage 2 (S2), fcl in rice and stl in *Arabidopsis* appeared fully as stage 3 (S3). The ratio of variable fluorescence to maximum fluorescence (Fv/Fm), which represents the maximum photochemical efficiency of PSII (Maxwell and Johnson, 2000; Hou et al., 2015), was significantly increased in the three stages of rice (Figure 1A). In contrast, unlike rice, *Arabidopsis* could germinate on 0.8% agar, but was arrested at the cotyledon stage without supplied nutrients (Figure 1B). In addition, the Fv/Fm was decreased during the growth (Figure 1B), suggesting that the nutrients stored in *Arabidopsis* seeds are insufficient to satisfy their normal growth and development. To ensure the normal growth of *Arabidopsis*, seedlings were grown on 1/2 MS medium containing 1% sucrose. No significant difference in Fv/Fm was found in the three stages of *Arabidopsis* (Figure 1C). Taken together, the results of the chlorophyll fluorescence assays suggested that differences exist in early growth and development between rice and *Arabidopsis* in PSII function.

To further visualize differences in the photosynthetic complexes in two model plants, we used qRT-PCR and immunoblotting to examine the abundance of subunits of the photosynthetic complexes including PSII: D2, CP47, PsbO, and PsbQ; and PSI: PsaD and PsaF. The Rubisco protein and Coomassie Blue staining were used as a loading control. The results showed that the protein expression of PSII subunits increased from cpt to fcl in rice. Conversely, there was no significant difference in the abundance of PSII and PSI subunits between the three stages in *Arabidopsis* (Figure 1D). The mRNA levels of these six genes were consisting with the protein level in both rice and *Arabidopsis* (Figure 1E). These findings suggest that chloroplast functional protein complexes gradually mature from cpt, icl to fcl during the early growth and development of rice. Photosynthesis of the chloroplast is also gradually improved in the above three stages. Conversely, chloroplast proteins and photosynthesis are fully developed at the cot stage of *Arabidopsis*. More importantly, we can use RNA expression levels to represent the protein expression levels of photosynthetic genes because their expression trend was consistent.

### Sequencing Data Summary

To systematically study the differences between rice and *Arabidopsis* during early growth and development, gene expression analyses were conducted by high-throughput RNA sequencing. A total of 54.9 Gb raw data for rice and 67.9 Gb raw data for *Arabidopsis* were generated. RNA sequencing was performed on the HiSeq X Ten platform, and we detected the expression of mRNA in all tissue samples. In detail, 91,035,666, 90,874,512, and 95,792,352 raw reads were obtained for rice coleoptile (cpt-A, B, and C, respectively); 86,766,618 and 95,714,768 raw reads were obtained for rice incomplete leaf (icl-A and B, respectively); and 88,510,902, 115,795,104, and 85,884,202 raw reads were obtained for rice first complete leaf (fcl-A, B, and C, respectively). A total of 99,311,856, 100,974.596, and 100,566,922 raw reads were obtained for *Arabidopsis* cotyledon (cot-A, B, and C, respectively); 85,926,770, 92,493,690, and 98,031,334 raw reads were obtained for *Arabidopsis* first true leaf (ftl-A, B, and C, respectively); 89,000,366, 125,026,956, and 98,057,458 raw reads were obtained for *Arabidopsis* second true leaf (stl-A, B, and C, respectively). The results of the RNA-Seq reads mapped on the reference are shown in Supplementary Table S2. Of the 17 library alignments, the unique mapping rate between clean reads and the reference genome was 93.96–98.83% (Supplementary Table S3). The three functional elements of gene proportions were 55.00–75.31% exon, 5.83–8.19% intron, 18.86–37.50% intergenic in rice and 83.98–88.71% exon, 4.76–8.39% intron, and 6.52–7.43% intergenic in *Arabidopsis* (Supplementary Table S4), and the normalized FPKM values for all mRNAs and lncRNAs in all samples are shown in Supplementary Table S5. The above results show that our sequencing results were reliable.

### Identification and Characterization of mRNA and Novel lncRNA in Rice and *Arabidopsis*

**FIGURE 2:**
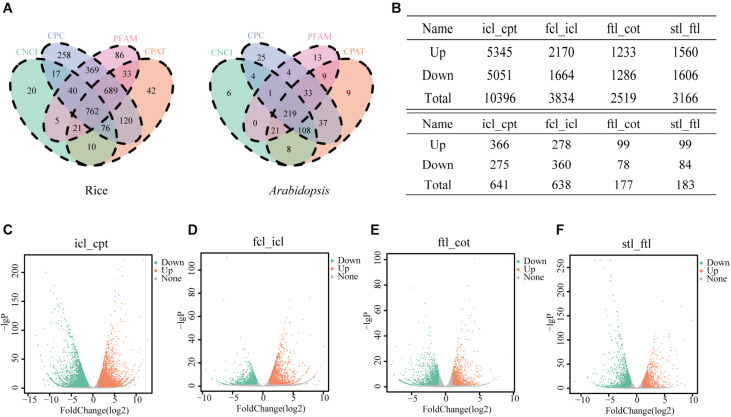
Differentially expressed unigenes and corresponding genes in each early growth developmental stage. **(A)** Three software CPC, CNCI, CPAT and a protein database pfam were used to predict lncRNAs, and the transcript was determined when the four methods were consistent in rice and *Arabidopsis*. **(B)** Number of differentially expressed genes obtained by comparisons between different stages and different lncRNAs in different stages. The genes were selected with “| log_2_ ratio| ≥ 1” and “*q* < 0.05.” **(C–F)** Volcanic map of differentially expressed genes in rice icl-vs.-cpt **(C)**, fcl-vs.-icl **(D)** and in *Arabidopsis* ftl-vs.-cot **(E)** and stl-vs.-ftl **(F)**. Volcano plot of the log_2_ fold change of gene expression levels (X-axis) against the –log_10_ *P*-value (Y-axis) comparing the two stages in rice and *Arabidopsis*. Splashes represent different genes. Gray splashes means genes without significant different expression. Orange splashes mean significantly upregulated genes. Green splashes mean significantly downregulated genes. The genes were selected with “| log_2_ ratio| ≥ 1” and “*q* < 0.05.”

In the present study, we collected three typical growth periods of rice and *Arabidopsis*. Using high-throughput sequencing, DEGs were detected in the leaves at different developmental stages. To study the basic features of lncRNAs in rice and *Arabidopsis*, lncRNAs were identified and compared with mRNAs. As a result, 762 novel transcripts in rice and 219 novel transcripts in *Arabidopsis* were identified as novel lncRNAs (Figure 2A). In addition, 43,148 known transcripts in rice and 25,481 in *Arabidopsis* were identified as mRNAs. Most identified lncRNAs had only 2–3 exons in their transcripts in both rice (Supplementary Figures S1A,E) and *Arabidopsis* (Supplementary Figures S1B,F) compared with mRNAs. The number of lncRNAs was notably small compared to the number of coding RNAs with 200–2000 nt transcripts in both rice (Supplementary Figures S1C,G) and *Arabidopsis* (Supplementary Figures S1D,H) compared with mRNAs. According to RNA-seq, the significantly dysregulated mRNAs and lncRNAs (|log_2_ ratio| ≥ 1, *q* < 0.05), including upregulated and downregulated mRNAs and lncRNAs, were found to be differentially expressed between icl-vs.-cpt and fcl-vs.-icl in rice; ftl-vs.-cot, stl-vs.-ftl in *Arabidopsis*. In rice, we identified 10396 and 3834 DEGs in the icl-vs.-cpt and fcl-vs.-icl comparisons, respectively (Figure 2B). Similarly, 2519 and 3166 DEGs were identified in the ftl-vs.-cot and stl-vs.-ftl comparisons, respectively, in *Arabidopsis* (Figure 2B). Similarly, according to RNA-seq, the significantly upregulated and downregulated lncRNAs (| log_2_ ratio| ≥ 1, *q* < 0.05) were found to be differentially expressed between the different stages (Supplementary Figure S2). In rice, we identified 641 and 638 DEGs in the icl-vs.-cpt and fcl-vs.-icl comparisons, respectively (Figures 2C,D). Similarly, 177 and 183 DEGs were identified in the ftl-vs.-cot and stl-vs.-ftl comparisons (Figures 2E,F), respectively, in *Arabidopsis*.

### GO Analysis of Differential Expressed mRNAs and Novel lncRNAs in Rice and *Arabidopsis*

To better understand the developmental pattern of the photosynthetic characteristics of early growth and development in rice and *Arabidopsis*, GO cluster analysis was conducted to further explore the biological processes enriched by the candidate mRNAs and lncRNAs. In rice, GO analysis for mRNAs revealed up- and downregulated genes in biological pathways, and “photosynthesis,” “photosynthesis, light reaction,” “photosynthetic electron transport in PSI,” and “photosynthesis, light harvesting in PSI” were clearly enriched in icl-vs.-cpt. “Photosynthesis,” “photosynthesis, dark reaction,” “photosynthesis, light reaction,” and “photosynthesis, light harvesting” were also clearly enriched in fcl-vs.-icl. In contrast, in *Arabidopsis*, no photosynthesis-related biological pathways were enriched in the ftl-vs.-cot and stl-vs.-ftl comparisons. Similarly, GO analysis for lncRNAs revealed that up- and downregulated genes in biological pathways in rice were “photosynthesis,” “photosynthesis, light reaction,” “photosynthesis, light harvesting,” “phototransduction,” and “red, far-red light phototransduction,” which were clearly enriched in icl-vs.-cpt. “Photosynthesis,” “photosynthesis, light reaction,” “photorespiration,” “photosynthesis, light harvesting,” and “chlorophyll metabolic process” were also clearly enriched in fcl-vs.-icl. In *Arabidopsis*, similar to the mRNAs, no photosynthesis-related biological pathways were enriched in the ftl-vs.-cot and stl-vs.-ftl comparisons (Supplementary Table S6). This result suggests that chloroplast develop and mature gradually in the three stages of early growth and development in rice, unlike the fully developed chloroplast in cotyledon in *Arabidopsis*.

### KEGG Analysis of Differential Expressed mRNAs and Novel lncRNAs in Rice and *Arabidopsis*

**FIGURE 3:**
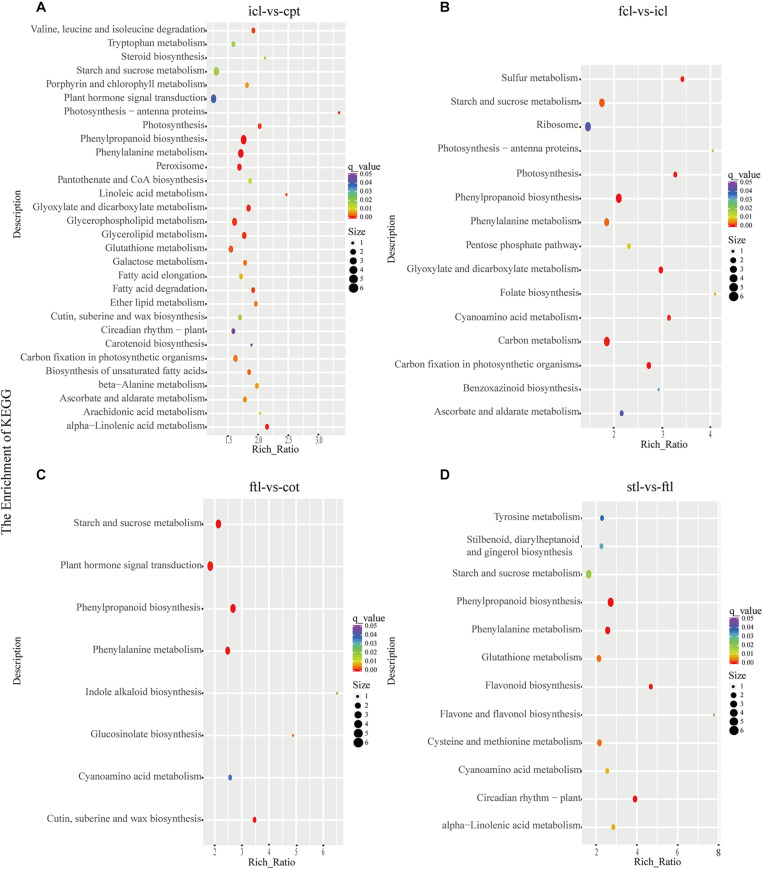
The mRNAs KEGG classifications of predicted target genes in different comparisons. KEGG classification of the predicted target genes in different comparisons of icl-vs.-cpt **(A)** and fcl-vs.-icl **(B)** in rice. KEGG classification of the predicted target genes in different comparisons of ftl-vs.-cot **(C)** and stl-vs.-ftl **(D)** in *Arabidopsis*. In both **(A,B),** the y-axis shows the pathway category, and the x-axis shows the richness factor. A larger richness factor indicates greater enrichment. The size of the point represents the number of related DEGs., and the color of the bubble represents the range of *q*-values.

**FIGURE 4:**
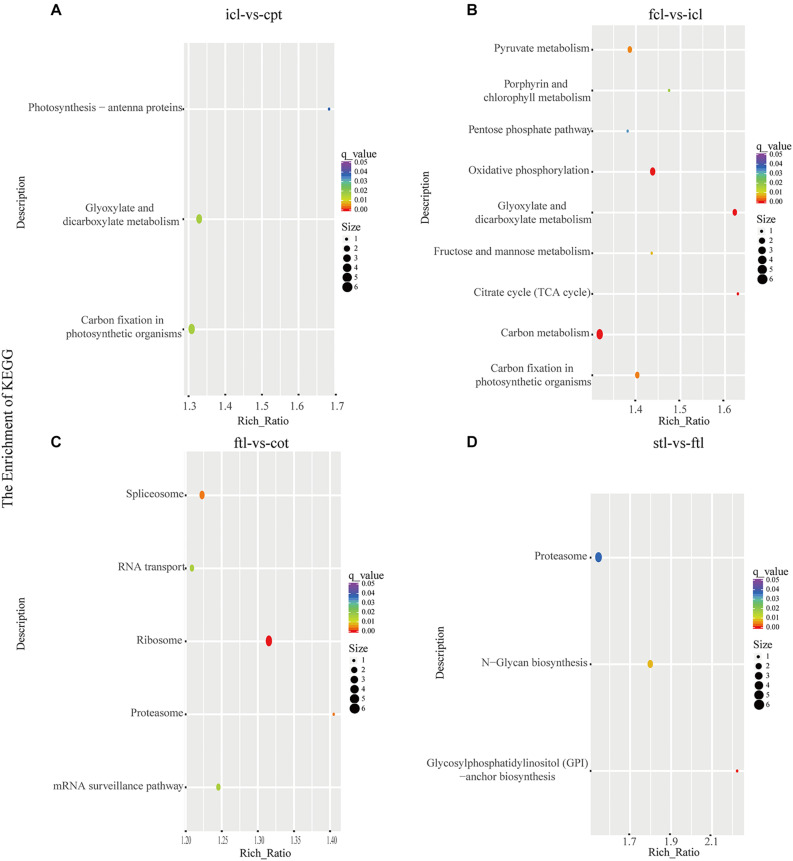
The lncRNAs KEGG classifications of predicted target genes in different comparisons. KEGG classification of the predicted target genes in different comparisons of icl-vs.-cpt **(A)** and fcl-vs.-icl **(B)** in rice. KEGG classification of the predicted target genes in different comparisons of ftl-vs.-cot **(C)** and stl-vs.-ftl **(D)** in *Arabidopsis*. In both **(A,B)**, the y-axis shows the pathway category, and the x-axis shows the richness factor. A larger richness factor indicates greater enrichment. The size of the point represents the number of related DEGs, and the color of the bubble represents the range of *q*-values.

At the same time, KEGG pathway analysis showed that mRNAs in the network of photosynthesis were enriched in “Photosynthesis antenna proteins,” “Photosynthesis,” and “Starch and sucrose metabolism” in icl-vs.-cpt (Figure 3A). “Photosynthesis,” “starch and sucrose metabolism,” and “photosynthesis – antenna proteins” were also significantly enriched in fcl-vs.-icl (Figure 3B). Similar to the GO analysis, in *Arabidopsis*, no photosynthesis-related biological pathways were enriched in the ftl-vs.-cot and stl-vs.-ftl comparisons (Figures 3C,D). Interestingly, the “starch and sucrose metabolism” pathway was clearly enriched in the ftl-vs.-cot and stl-vs.-ftl comparisons (Figures 3C,D). Similarly, KEGG analysis for lncRNAs revealed that up- and downregulated photosynthesis-related genes in rice were “Photosynthesis – antenna proteins,” “Carbon fixation in photosynthetic organisms” in icl-vs.-cpt (Figure 4A). “Carbon fixation in photosynthetic organisms” and “porphyrin and chlorophyll metabolism” in fcl-vs.-icl (Figure 4B). Additionally, “Carbon metabolism” and “Citrate cycle (TCA cycle)” were clearly enriched in fcl-vs.-icl (Figure 4B). This finding suggests that the accumulation of photosynthesis products increased gradually from cpt to fcl. Similar to the GO analysis in *Arabidopsis*, no photosynthesis-related biological pathways were enriched in the ftl-vs.-cot and stl-vs.-ftl comparisons (Figures 4C,D).

The KEGG analysis combined with GO analysis of DEGs indicated that there are great differences in the expression pattern of chloroplast-related genes and the function of chloroplast form integrity during early development between rice and *Arabidopsis*. Photosynthetic genes and functions develop and mature gradually in rice and fully develop when exposed to light in *Arabidopsis* in the three stages of early growth and development. More importantly, lncRNAs might play important roles in chloroplast development by regulating these coherent target genes. The difference in chloroplast development between rice and *Arabidopsis* is largely consistent with the mRNA level.

### Different Patterns of Photosynthesis Metabolism in Early Development

**FIGURE 5:**
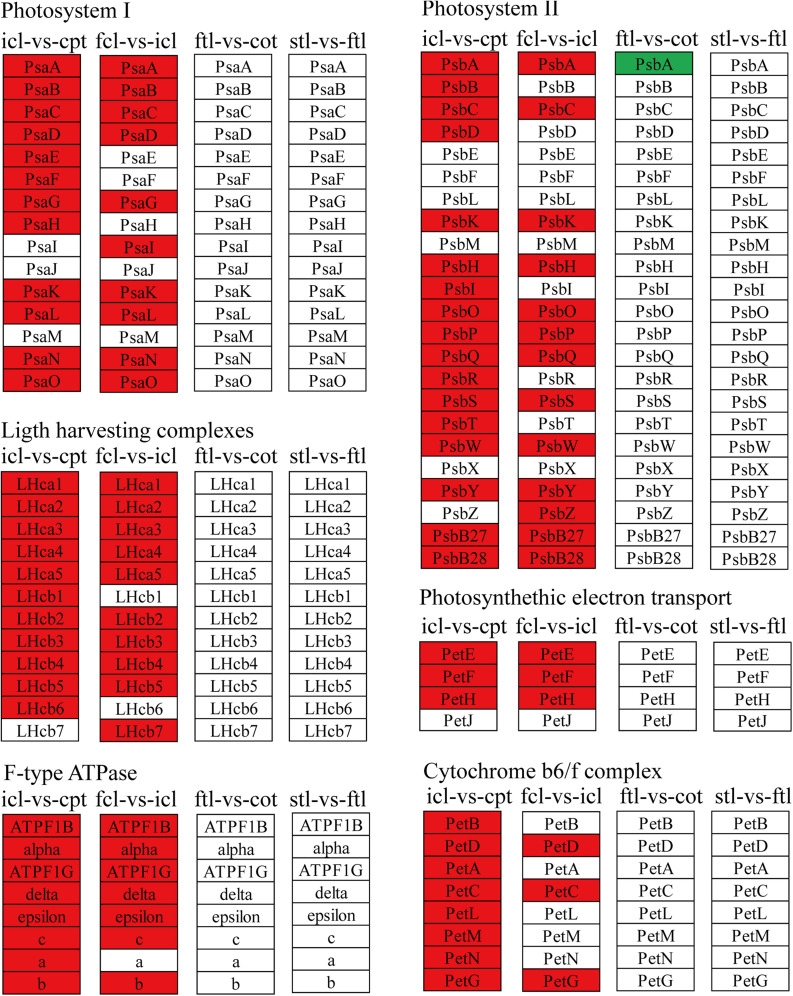
The mRNAs KEGG classifications of photosynthetic genes in different comparisons. KEGG classification of the photosynthetic genes in different comparisons of icl-vs.-cpt and fcl-vs.-icl in rice, ftl-vs.-cot and stl-vs.-ftl in *Arabidopsis*. The change of expression amount was expressed by color change, the red color represented upregulated expression annotated to the KEGG Orthology (KO) system, the green color represented downregulated expression annotated to the KO system. The genes were selected with “| log_2_ ratio| ≥ 1” and “*q* < 0.05.”

**FIGURE 6:**
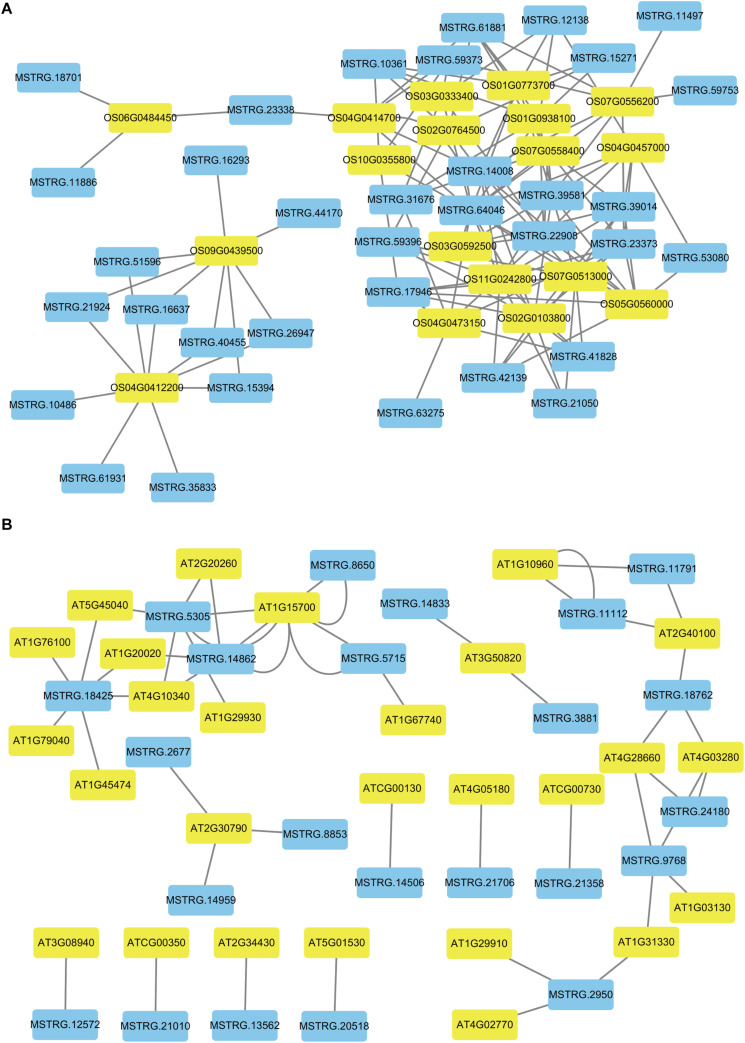
The networks of novel lncRNAs and target mRNAs of DEGs related to photosynthesis. **(A)** Co-expression network analysis of novel lncRNAs and target mRNAs in rice. **(B)** Co-expression network analysis of novel lncRNAs and target mRNAs in *Arabidopsis*. The blue color indicated novel lncRNAs and the yellow color indicated target genes for novel lncRNAs.

**FIGURE 7:**
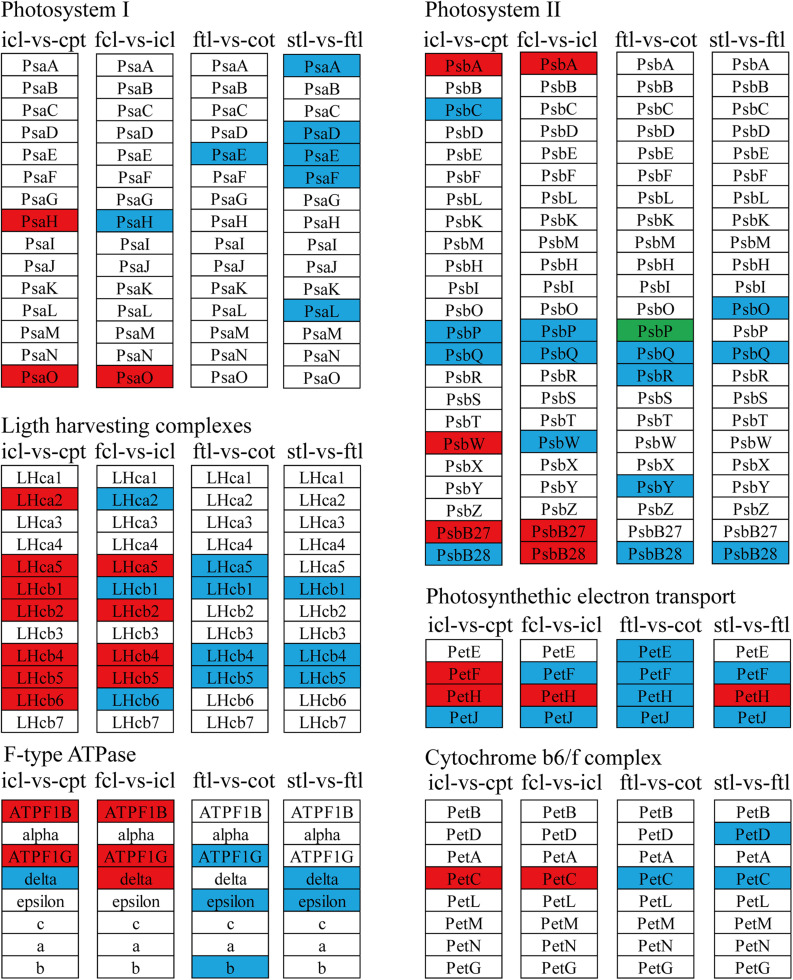
The lncRNAs KEGG classifications of photosynthetic genes in different comparisons. KEGG classification of the photosynthetic genes in different comparisons of icl-vs.-cpt and fcl-vs.-icl in rice, ftl-vs.-cot, and stl-vs.-ftl in *Arabidopsis*. The change of expression amount was expressed by color change, the red color represented upregulated expression annotated to the KEGG Orthology (KO) system, the green color represented downregulated expression annotated to the KO system, blue color indicates that the target gene annotated to the KO system is not significantly up- and downregulated. The genes were selected with “| log_2_ ratio| ≥ 1” and “*q* < 0.05.”

To better describe the differences in gene expression at different stages between rice (from cpt to fcl) and *Arabidopsis* (from cot to stl). We studied DEGs related to photosynthesis in rice and *Arabidopsis*. The up-regulated genes in fcl compared to icl were distributed in PSII, PSI, cytochrome b6/f complex, photosynthetic electron transport and F-type ATPase (Figure 5). The functions of lncRNAs are executed on coding genes via *cis*- or *trans*-regulation. Different expression novel lncRNAs targeted photosynthetic genes in rice and *Arabidopsis* were showed in Figure 6 and Supplementary Table S7. As showed in Figure 6, one novel lncRNA was correlated with several mRNAs and one mRNA was co-expressed with more than one novel lncRNA, further supporting the notion of inter-regulatory relationships. The novel lncRNAs targeted photosynthetic genes also showed upregulated chloroplast-associated genes, including *PsbA*, *PsbW*, *Psb27*, *PsaH*, *PsaO*, *PetC*, *PetF*, *PetH*, *ATPF1B*, and *ATPF1G* in icl-vs.-cpt, and *PsbA*, *Psb27*, *Psb28*, *PsaO*, *PetC*, *PetH*, *ATPF1B*, *ATPF1G*, and *ATPF1D* in fcl-vs.-icl. In contrast, for *Arabidopsis*, KEGG pathway analysis of novel lncRNAs targeted photosynthetic genes showed no significant difference in chloroplast-associated genes in the three stages (Figure 7). To validate the RNA-Seq data, DEGs and differentially expressed mRNAs and lncRNAs related to photosynthesis were selected in rice and *Arabidopsis* at the three stages (Supplementary Table S8). The qRT-PCR expression patterns of mRNAs and lncRNAs genes were consistent with the sequencing results (Supplementary Figure S3). In addition, we performed qRT-PCR analysis of the genes mentioned in this paper, and further analysis of the correlation between RNA Seq and q-RT PCR results. As shown in Supplementary Figure S4 and Supplementary Table S9, the expression trends of all 17 genes from qRT-PCR and RNA-seq analyses including mRNAs and lncRNAs were largely consistent (Pearson’s correlation coefficient *R*^2^ = 0.83 in rice and 0.91 in *Arabidopsis*), indicating that our sequencing results were reliable.

The light-harvesting chlorophyll protein complex (LHC) is also enriched in mRNA and lncRNA DEGs in rice. For icl-vs.-cpt, *LHCA2*, *LHCA5*, *LHCB1*, *LHCB2*, *LHCB4*, *LHCB5*, and *LHCB6* were upregulated by KEGG in both mRNA and lncRNA DEGs (Figures 5, 7). For fcl-vs.-icl, *LHCA2*, *LHCA5*, *LHCB1*, *LHCB2*, *LHCB4*, and *LHCB5* were enriched in mRNA; *LHCA5*, *LHCB2*, *LHCB4*, and *LHCB5* were upregulated in lncRNA (Figures 5, 7). In contrast, there was no significant difference in LHC genes in ftl-vs.-cot in either mRNA or lncRNA DEGs (Figures 5, 7); *LHCB1* was upregulated and *LHCB4* was downregulated in mRNA DEGs for stl-vs.-ftl, and there was no significant difference in LHC genes in lncRNAs for stl-vs.-ftl (Figures 5, 7).

### Different Regulatory Styles of Assembly Factors for PSI and PSII in Early Development

**FIGURE 8:**
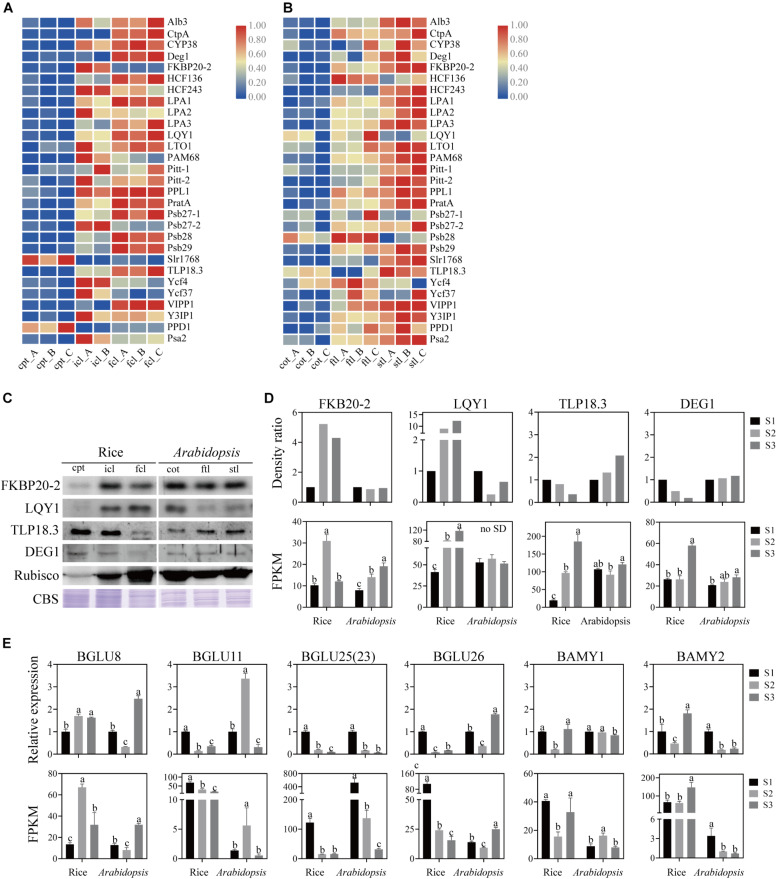
Analysis of PSII and PSI assembly factors proteins and genes, starch and sucrose metabolism pathways marker genes of rice and *Arabidopsis* in three stages. Heatmap of the expression of photosystems assembly factors in rice **(A)** and *Arabidopsis* **(B)** at different stages. The gene expression levels are shown in different colors indicated by the scale bar. **(C)** Analysis of photosystems assembly factor proteins in rice and *Arabidopsis* at different stages. 16 μg total proteins were separated by 12% SDS PAGE. And immunoblots were performed using antibodies as indicated. CBS, Coomassie Blue Staining. **(D)** The density ratio levels of photosystems assembly factor proteins in **(C)** and RNA-seq-based gene expression values (FPKM) of relavent genes in rice and *Arabidopsis*. Data was presented as the means ± SD of three biological replicates. a, b, and c indicate significant differences between the three stages according to Tukey’s test. **(E)** The relative expression levels of starch and sucrose metabolism pathways marker genes measured by real-time RT-PCR and FPKM of relavent genes in rice and *Arabidopsis*. Data was presented as means ± SD of three biological replicates. a, b, and c indicate significant differences between the three stages according to Tukey’s test.

The assembly of core subunits of PSII is regulated by the expression of nuclear genes and transported to plastids, and PSI assembly factors are encoded by both plastid and nuclear genes (Yang et al., 2015). We found that the expression values of the assembly factors of PSI and PSII were different between rice and *Arabidopsis* (Figures 8A,B and Supplementary Table S10). We used four genes as examples to explore the similarities and differences in PSI and PSII assembly during early development in rice and *Arabidopsis* (Figures 8C,D). The mRNA level of *FKBP20-2* was upregulated in icl-vs.-cpt and downregulated in fcl-vs.-icl in rice based on the sequencing results. In contrast, the expression levels were upregulated in cot-vs.-ftl and stl-vs.-ftl in *Arabidopsis*. To further visualize changes in the protein expression level, we used immunoblotting to examine the abundance of the assembly factors of three stages in rice and *Arabidopsis*. The results showed upregulation in icl-vs.-cpt and downregulation in fcl-vs.-icl in rice with no significant difference in cot-vs.-ftl and stl-vs.-ftl in *Arabidopsis*. For FKBP20-2, the RNA expression levels were consistent with the protein expression levels in rice for three stages but did not appear in *Arabidopsis*. The mRNA levels of *LQY1* were upregulated in icl-vs.-cpt and fcl-vs.-icl in rice, upregulated in cot-vs.-ftl and downregulated in stl-vs.-ftl in *Arabidopsis*. The immunoblots showed upregulation in icl-vs.-cpt and fcl-vs.-icl in rice, downregulation in cot-vs.-ftl and upregulation in stl-vs.-ftl in *Arabidopsis*. For LQY1, the RNA expression levels were consistent with the protein expression levels in rice and for three stages but did not appear in *Arabidopsis*. The mRNA levels of *TLP18.3* were upregulated in icl-vs.-cpt and fcl-vs.-icl in rice, downregulated in cot-vs.-ftl and upregulated in stl-vs.-ftl in *Arabidopsis*. The immunoblots showed downregulation in icl-vs.-cpt and fcl-vs.-icl in rice and upregulation in cot-vs.-ftl and stl-vs.-ftl in *Arabidopsis*. For TLP18.3, the RNA expression levels were not consistent with protein expression levels in rice and for three stages, but they appeared in *Arabidopsis*. The mRNA level of *Deg1* was not significantly different in icl-vs.-cpt and was upregulated in fcl-vs.-icl in rice with no significant difference in cot-vs.-ftl and upregulated in stl-vs.-ftl in *Arabidopsis*. The immunoblots showed downregulated icl-vs.-cpt and fcl-vs.-icl in rice, with no significant difference in cot-vs.-ftl and stl-vs.-ftl in *Arabidopsis*. For Deg1, the RNA expression levels were not consistent with the protein expression levels in rice and *Arabidopsis* for three stages. Taken together, these results indicate that assembling the individual subunits to form PSII and PSI may not occur at the transcriptional level.

### Different Patterns of Starch and Sucrose Metabolism in Early Development

Starch is a storage carbohydrate widely synthesized in plants. Starch serves as an important store of carbon that fuels plant metabolism and growth when they are unable to photosynthesize. The “starch and sucrose metabolism” terms were all clearly enriched among the DEGs in rice and *Arabidopsis*. To test the similarities and differences in expression patterns, four β-glucosidase (EC:3.2.1.21) genes, α-amylase (EC:3.2.1.1) gene and β-amylase (EC:3.2.1.2) gene were analyzed. Similarly, the qRT-PCR expression patterns of mRNA and lncRNA genes were consistent with the sequencing results (Figure 8E). These results indicate that although DEGs of starch and sucrose metabolism pathways are expressed both in rice and *Arabidopsis*, there are significant differences in the expression trend of key genes between the two species.

## Discussion

In this study, significant advances have been made in understanding the photosynthetic gene functions from coleoptile, incomplete leaf to first complete leaf in rice, from cotyledon, first true leaf to second true leaf in *Arabidopsis*. Many protein coding genes have been identified by traditional genetic and molecular analysis combined with recent genomic studies, which can act as positive or negative regulators of photomorphogenesis in seedlings under different light conditions (Jiao et al., 2007; Chen and Chory, 2011; Li et al., 2012). lncRNAs are an important class of non-coding RNAs (ncRNAs) which have been proved to have multiple functions in eukaryotes recently (Wang and Chang, 2011; Guttman and Rinn, 2012; Geisler and Coller, 2013). lncRNAs have been systematically identified or predicted in *Arabidopsis*, rice, wheat, and maize (De Lucia and Dean, 2011; Kim and Sung, 2012; Zhang and Chen, 2013). The non-coding RNA HIDDEN TREASURE 1 (HID1) is a positive regulator of photomorphogenesis (Wang et al., 2014a). lncRNAs can regulate photoperiod-sensitive male sterility (PSMS) in rice (Ding et al., 2012). Chloroplasts are green plastids found in land plants, algae, and some protists. Chloroplasts are the only site of photosynthesis in these cells. Therefore, chloroplasts are responsible for most of the world’s primary productivity (Jarvis, 2011). In our study, chlorophyll fluorescence (Fv/Fm) from icl, cpt to fcl increased progressively in rice, indicating that the development of rice chloroplasts matured, in turn, from coleoptile to true leaf. In addition, western blotting also indicated that the development and maturation process of rice chloroplasts exhibits gradual maturation. In contrast, in *Arabidopsis*, the Fv/Fm ratio from cot, first true leaf to second true leaf was almost unchanged, and proteins analysis showed the same result. Therefore, there were significant differences in the early development of chloroplasts between rice and *Arabidopsis*.

In this study, we investigated mRNA-dependent mRNA-lncRNA interactions in different stages for early growth and development using bioinformatics approaches. Chloroplast is an essential semiautonomous organelle, which plays an important role in photosynthesis, carbon fixation, oxygen production, energy conversion, starch production, amino acid synthesis, fatty acid synthesis, pigment synthesis, hormone synthesis and sulfur and nitrogen metabolism (Neuhaus and Emes, 2000; Jarvis and Lopez-Juez, 2013; Xu et al., 2016; Wang et al., 2018). For rice, it is essential to know the development of photosynthetic genes. The gene expression of rice coleoptile and incomplete chloroplasts was significantly lower than that of the first complete leaf. This finding indicates that the photosynthetic genes are fully developed during the first complete development period. In contrast, in *Arabidopsis*, the expression of photosynthetic genes in cotyledons was similar to that in true leaves. This finding indicates that the photosynthetic genes have been developed in the cotyledon stage. In our study, we found that the function of the first complete leaf of rice is similar to that of the cotyledon of *Arabidopsis* in chloroplast development.

GO cluster analysis of rice showed that in the three stages of early development growth and development, the DEGs in biological pathways were mainly related to photosynthesis both in mRNA and lncRNA. Additionally, KEGG pathway analysis showed that mRNAs in the photosynthesis network were enriched in “Photosynthesis antenna proteins,” “Photosynthesis,” and “Starch and sucrose metabolism” in icl-vs.-cpt. “Photosynthesis,” “Starch and sucrose metabolism,” and “Photosynthesis antenna proteins” in fcl-vs.-icl. In *Arabidopsis*, no photosynthesis-related biological pathways were enriched in the ftl-vs.-cot and stl-vs.-ftl comparisons. Interestingly, the “starch and sucrose metabolism” pathway was clearly enriched in the ftl-vs.-cot and stl-vs.-ftl systems. KEGG analysis of lncRNAs revealed that up- and downregulated photosynthesis-related genes in rice were “Photosynthesis – antenna proteins,” “Carbon fixation in photosynthetic organisms” in icl-vs.-cpt. “Carbon fixation in photosynthetic organisms” and “porphyrin and chlorophyll metabolism” in fcl-vs.- icl (Figure 4B). Additionally, “Carbon metabolism” and “Citrate cycle (TCA cycle)” were clearly enriched in fcl-vs.-icl. In *Arabidopsis*, no photosynthesis-related biological pathways were enriched in the ftl-vs.-cot and stl-vs.-ftl comparisons.

The main DEGs were chloroplast development and the carbon cycle pathway in early rice growth and development. Sucrose and starch contents due to alterations of the allocation pattern of photosynthetic fixed carbon (Higuchi et al., 2015; Pilkington et al., 2015). Temporary starch exists in cells with photosynthetic capacity, synthesized in the chloroplast during the day, and degraded at night to provide carbon for non-photosynthetic metabolism (Lunn, 2007). Leaf starch and sucrose allocation is a well-regulated process in carbohydrate metabolism. The amount of starch synthesis seems to be in harmony with the length of the dark period (Cordenunsi-Lysenko et al., 2019). Starch-sucrose distribution is affected by plant development and sink activity, as well as environmental factors such as light intensity, temperature and photoperiod length (Pilkington et al., 2015). This finding indicates the involvement of lncRNAs in the regulation of chloroplast-related biological processes in rice. In *Arabidopsis*, the KEGG analysis of differentially expressed mRNAs revealed that the main enrichments in ftl-vs.-cot were phenylpropanoid biosynthesis, starch and sucrose metabolism, phenylalanine metabolism and cutin, suberine and wax biosynthesis; phenylpropanoid biosynthesis, flavonoid biosynthesis, circadian rhythm-plant and phenylalanine metabolism in stl-vs.-ftl. The KEGG analysis of differentially expressed lncRNAs showed that the main enrichments were ribosome in ftl-vs.-cot and glycosylphosphatidylinositol (GPI)-anchor biosynthesis in stl-vs.-ftl. This finding suggests that lncRNAs may not participate in chloroplast development and related biological processes in *Arabidopsis*. These results indicate that there are important differences between monocotyledon rice and dicotyledon *Arabidopsis* in early growth and development. In addition, we found that the plant could not grow without supplied nutrients for *Arabidopsis* (Figure 1), but rice grew normally under these conditions. It has been proven that there are differences between rice and *Arabidopsis* in early growth and development.

The chloroplast of eukaryotic cells is developed from an ancient endosymbiont related to cyanobacteria, which gives plants the ability of oxygenic photosynthesis (Gould et al., 2008; Nickelsen and Rengstl, 2013). The chloroplast is a semiautonomous organelle. The chloroplast has retained some genes, and different subunits of organellar protein complexes are derived from different genetic systems. PSII assembly factors are all encoded by the nucleus and transported to chloroplasts, and PSI assembly factors are encoded by nuclear genes and photosynthetic genes. From the mRNA sequencing data, we concluded that the assembly processes of PSI and PSII are not exactly the same. The expression trends of the four assembly genes for PSI and PSII differed from those of rice and *Arabidopsis*. FKBP20-2 participates in the assembly of the PSII supercomplexes in the chloroplast thylakoid lumen (Lima et al., 2006). LQY1 protein is involved in PSII photo-repair and reassembly of PSII complexes (Lu et al., 2011). TLP18.3 protein was involved in PSII regulating repair, turnover of damaged D1 and reassembly of PSII (Sirpio et al., 2007). Deg1 assists the assembly of the PSII complex through interaction with the PSII reaction center D2 protein (Sun et al., 2010). Together with the western blot results, FKB20-2 and LQY1 had the same expression pattern, but TLP18.3 and DEG1 had the opposite expression pattern of the three stages in rice. Similarly, in *Arabidopsis*, except for DEG1, FKB20-2, LQY1, and TLP18.3 had the opposite expression pattern of the three stages. The qRT–PCR and western blot results indicated that the PSI and PSII assembly were not correlated with its transcription level in the two species. Moreover, different expression levels of assembly factors in rice and *Arabidopsis* lead to different functions of PSI and PSII.

We take starch and sucrose metabolism as an example. The four β-glucosidase (EC:3.2.1.21) genes, α-amylase (EC:3.2.1.1) gene and β-amylase (EC:3.2.1.2) gene were analyzed. *BGLU25*/*BGLU23* represents an independent class of six thioglucoside glucohydrolases (Nakano et al., 2017), *BGLU8* encodes a putative beta glucosidase (Wang et al., 2017), *BGLU26* encodes an atypical myrosinase (Matern et al., 2019), *BGLU11* encodes beta glucosidase 11. *BAMY1* is involved in diurnal starch degradation in guard cells (Zanella et al., 2016), *AMY2/BAMY2* was a mixed-function OSC capable of synthesizing both β-amyrin and lupeol (Iturbe-Ormaetxe et al., 2003). In rice, β-glucosidase (EC:3.2.1.21), as a carbohydrate with biological functions, participates in energy acquisition, cell wall extension and signal transduction through selective hydrolysis of glycoside bonds in plants and plays an important role in the defense of pathogens (Davies and Henrissat, 1995; Mizutani et al., 2002; Barleben et al., 2005; Lee et al., 2006; Morant et al., 2008); alpha-amylase (EC:3.2.1.1) acts on the alpha-1,4-glycoside bond of starch but cannot cut the alpha-1,6-glycoside bond at the branch of amylopectin, thereby hydrolyzing the substrate (Wang et al., 2006); glucan 1,3-β-glucosidase (EC:3.2.1.58) is a growth-specific cell wall polysaccharide that exists in the endosperm of members of the Gramineae family (grasses) and commercially important grains (such as barley, rice, and wheat) among the main components of the cell wall (Hwang du et al., 2007). Those biological processes were significantly downregulated; endoglucanase (EC:3.2.1.4) hydrolyzed cellulose randomly in amorphous regions, producing oligosaccharides of different lengths, cellobiose, and glucose (Klippel et al., 2019), and showed significant upregulation in icl-vs.-cpt; in addition, no significant difference in fcl-vs.-icl. In *Arabidopsis*, the main upregulated gene is β-glucosidase (EC:3.2.1.21), and the downregulated gene is α-amylase (EC:3.2.1.1) in ftl-vs.-cot. The upregulated gene is β-glucosidase (EC:3.2.1.21), and the downregulated gene is β-amylase (EC:3.2.1.2), which hydrolyzes every other α-1,4-glycoside bond from the non-reductive end of starch substrate once, and the hydrolysate is maltose unit (Wang et al., 2006) in stl-vs.-ftl. In addition, no lncRNA DEGs showed significant differences in the three stages of the starch and sucrose metabolism pathways.

## Conclusion

mRNA and lncRNAs sequencing revealed that photosynthesis was gradually established from coleoptile, incomplete leaf to complete leaf in rice and was fully functional in cotyledon of *Arabidopsis*. In summary, we examined the gene expression patterns of mRNAs and lncRNAs at different developmental stages of early growth and development in rice and *Arabidopsis*. In our study, functional modes were revealed for mRNAs and lncRNAs, the main DEGs enriched in multiple biological processes, such as photosynthetic genes and photosynthetic carbon assimilation genes. We found that the chloroplast protein from the coleoptile, incomplete leaf and first complete leaf gradually matured in rice. In contrast, in *Arabidopsis*, the maturation of chloroplast protein was immediately complete at the cotyledon stage. The results showed that monocotyledons and dicotyledons (at least in rice and *Arabidopsis*) differed greatly in photosynthetic mechanisms. In conclusion, our studies revealed whole genome expression profiles of mRNAs and lncRNAs related to early growth and development in rice and *Arabidopsis*, thereby providing important evidence for further research on the molecular mechanisms of early growth and development.

## Data Availability Statement

The datasets presented in this study can be found in online repositories. The names of the repository/repositories and accession number(s) can be found below: https://www. ncbi.nlm.nih.gov/, SRR10907568–SRR10907573; https://www.ncbi.nlm.nih.gov/, SRR10913120-SRR10913123; https://www.ncbi. nlm.nih.gov/, SRR10913307–SRR10913312; https://www.ncbi.nlm.nih.gov/, SRR10914245–SRR10914250; https://www.ncbi.nlm.nih.gov/, SRR10914757–SRR10914762; https://www.ncbi.nlm.nih.gov/, SRR10915176–SRR10915181.

## Author Contributions

YS and XH designed the research and wrote the manuscript. YS and JC performed the research. All authors analyzed the data and read and approved the manuscript.

## Conflict of Interest

The authors declare that the research was conducted in the absence of any commercial or financial relationships that could be construed as a potential conflict of interest.

## Footnotes

1 http://www.genome.jp/kegg/

2 https://trace.ncbi.nlm.nih.gov/Traces/sra/sra.cgi?view=run_browser

## Supplementary Material

FIGURE S1 The qualitative analysis of novel lncRNAs and the relationship about lncRNAs and mRNAs. The qualitative analysis of novel lncRNAs and the relationship about lncRNAs and mRNAs. Novel lncRNA exon distribution in rice **(A)** and *Arabidopsis* **(B)**; Novel lncRNA length distribution in rice **(C)** and *Arabidopsis* **(D)**; Exon number contrast of lncRNA vs. mRNA in rice **(E)** and *Arabidopsis* **(F)**; The length contrast of lncRNA vs. mRNA in rice **(G)** and *Arabidopsis* **(H)**.

FIGURE S2 Cluster map of differential genes rice and *Arabidopsis*. Cluster map of differential genes rice mRNAs **(A)**, rice lncRNAs **(B)**, *Arabidopsis* mRNAs **(C),** and *Arabidopsis* lncRNAs **(D)** The change of expression amount was expressed by color change, the yellow color represented higher expression, and the blue color represented lower expression.

FIGURE S3 The RNA-seq data and qRT-PCR validation of lncRNAs and their target protein-coding genes. The relative expression levels and RNA-seq-based gene expression values (FPKM) of six lncRNAs and their target protein-coding genes were shown. A lncRNA can have one or more target protein-coding genes. The bars denote the standard deviation.

FIGURE S4 Verification of the transcriptome results through the experiments of qRT-PCR. The relationships between qRT-PCR and RNA-seq. Values were the log_2_ ratio (FPKM ratio by RNA-Seq) for genes in rice **(A)** and *Arabidopsis* **(B)**. The determine coefficient (*R*^2^) was indicated in the figure. All qRT-PCR reactions were performed in three biological replicates.

TABLE S1 The primers used for quantitative real-time PCR.

TABLE S2 Summary of reads mapping to rice and *Arabidopsis* genome in three developmental stages.

TABLE S3 Summary of reads quality of rice and *Arabidopsis* in three developmental stages.

TABLE S4 Number and proportion of unique alignment sequences on three functional elements of gene (exon, intron and intergenic) in rice and *Arabidopsis*.

TABLE S5 The normalized FPKM values for all lncRNAs and mRNAs in all samples in rice and *Arabidopsis*.

TABLE S6 Gene ontology of biological pathways enriched in the transgenic lines based on the up- or down-regulated genes.

TABLE S7 Trans and *cis* target photosynthetic genes of novel lncRNAs in rice and *Arabidopsis*.

TABLE S8 The coherent target genes of differentially expressed lncRNAs in all comparisons.

TABLE S9 The relationships between qRT-PCR analysis for validation of RNA seq data analysis in rice and *Arabidopsis*.

TABLE S10 Summary of described PSII assembly factors in all samples in rice and *Arabidopsis*.

## Abbreviations

CNCI: Coding-Non-Coding Index
cot: cotyledon
CPAT: Coding Potential Assessment Tool
CPC: Coding Potential Calculator
cpt: coleoptile
DEGs: differentially expressed genes
fcl: first complete leaf
ftl: first true leaf
GO: Gene Ontology
icl: incomplete leaf
KEGG: Kyoto Encyclopedia of Genes and Genomes
KO: KEGG Orthology
lncRNA: long non-coding RNA
mRNA: messenger RNA
PS I: photosystem I
PS II: photosystem II
stl: second true leaf.
