# Supplementary figures and images for: Similarities and Differences of Photosynthesis Establishment Related mRNAs and Novel lncRNAs in Early Seedlings (Coleoptile/Cotyledon vs. True Leaf) of Rice and Arabidopsis
Source: Front Genet. 2020 Sep 8;11:565006. doi: 10.3389/fgene.2020.565006 (PMC7506105; doi:10.3389/fgene.2020.565006)

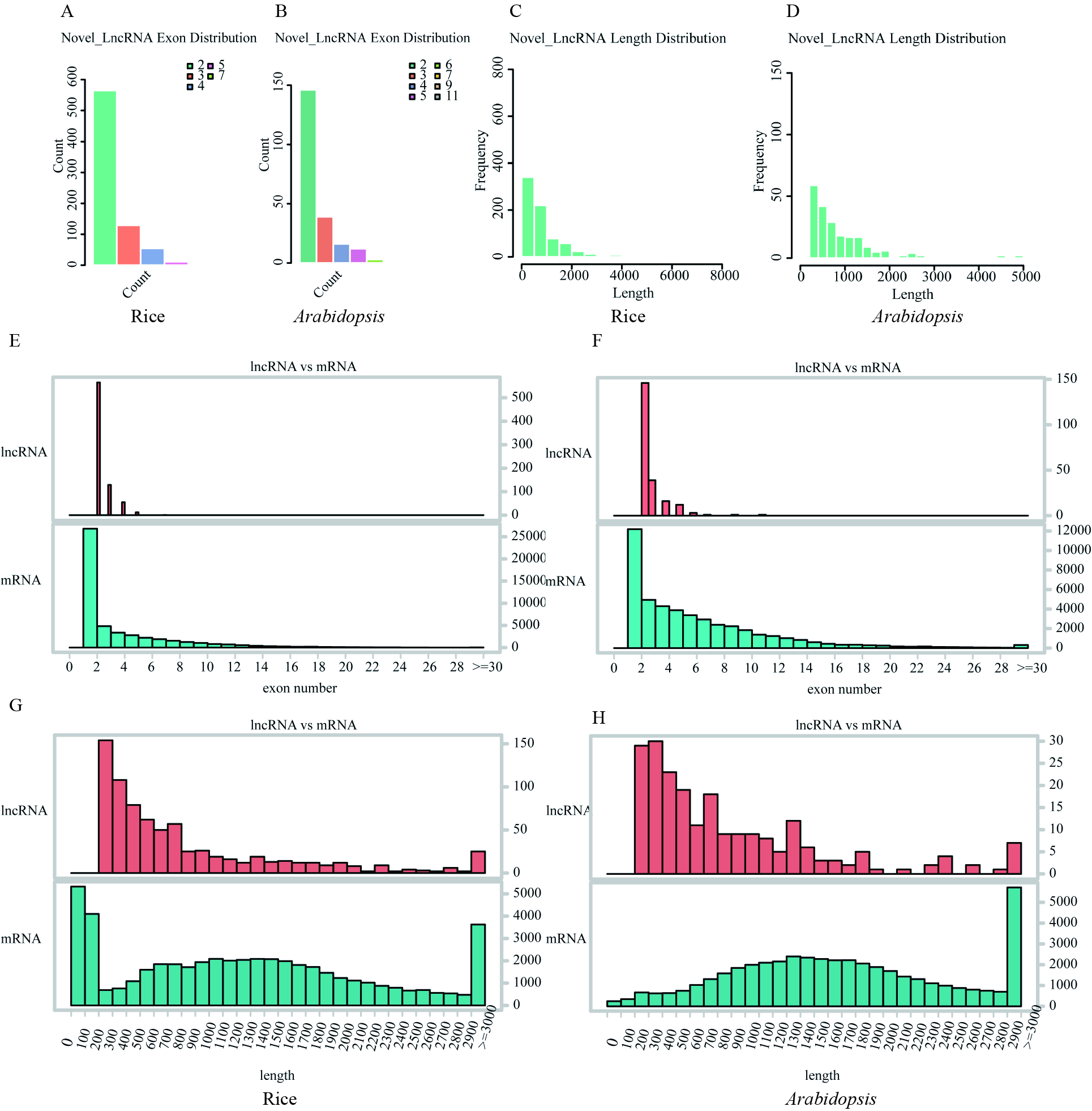

Supplement: FIGURE S1 — The qualitative analysis of novel lncRNAs and the relationship about lncRNAs and mRNAs. The qualitative analysis of novel lncRNAs and the relationship about lncRNAs and mRNAs. Novel lncRNA exon distribution in rice (A) and Arabidopsis (B); Novel lncRNA length distribution in rice (C) and Arabidopsis (D); Exon number contrast of lncRNA vs. mRNA in rice (E) and Arabidopsis (F); The length contrast of lncRNA vs. mRNA in rice (G) and Arabidopsis (H). [file Image_1.TIF]

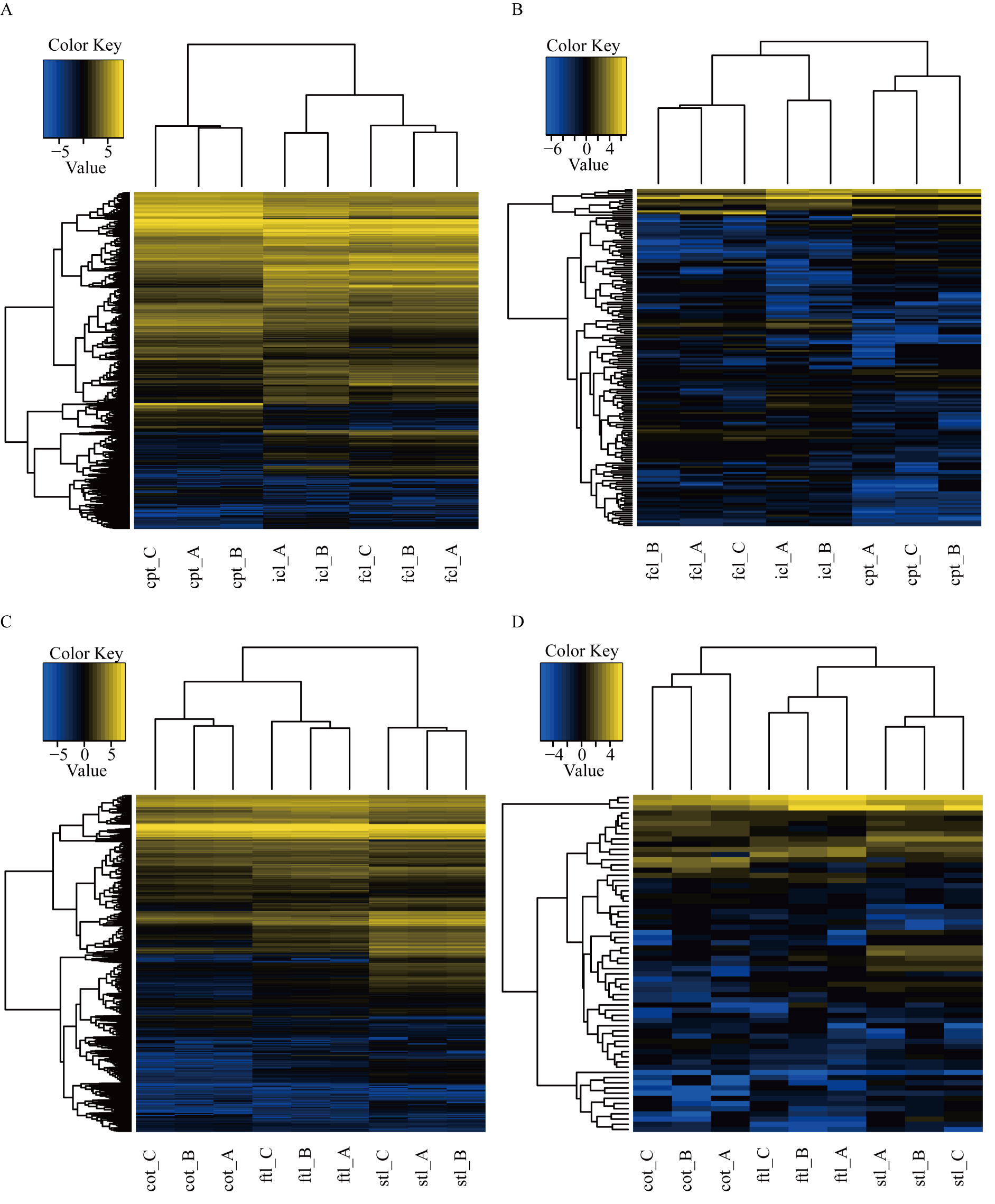

Supplement: FIGURE S2 — Cluster map of differential genes rice and Arabidopsis. Cluster map of differential genes rice mRNAs (A), rice lncRNAs (B), Arabidopsis mRNAs (C), and Arabidopsis lncRNAs (D) The change of expression amount was expressed by color change, the yellow color represented higher expression, and the blue color represented lower expression. [file Image_2.TIF]

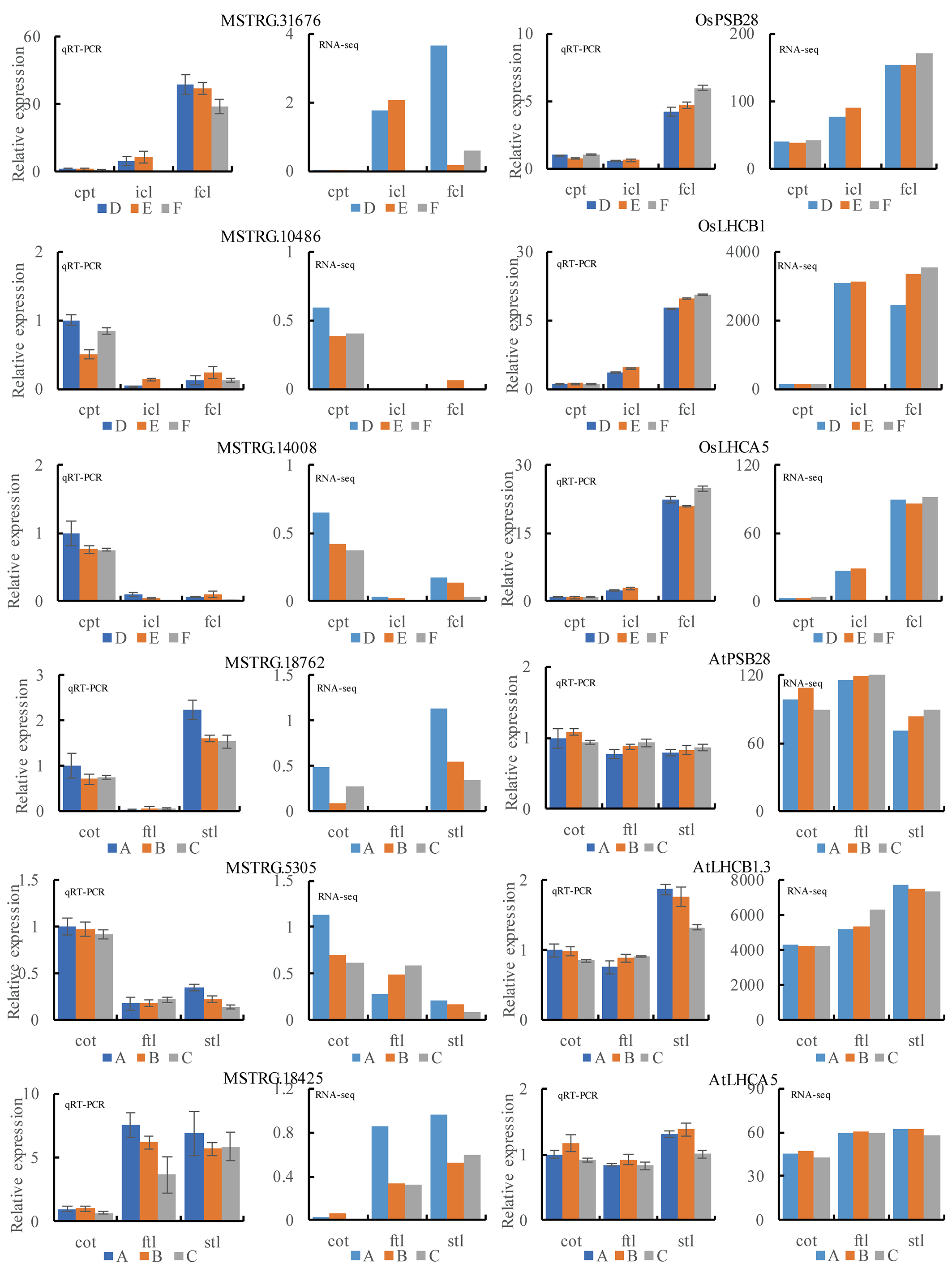

Supplement: FIGURE S3 — The RNA-seq data and qRT-PCR validation of lncRNAs and their target protein-coding genes. The relative expression levels and RNA-seq-based gene expression values (FPKM) of six lncRNAs and their target protein-coding genes were shown. A lncRNA can have one or more target protein-coding genes. The bars denote the standard deviation. [file Image_3.TIF]

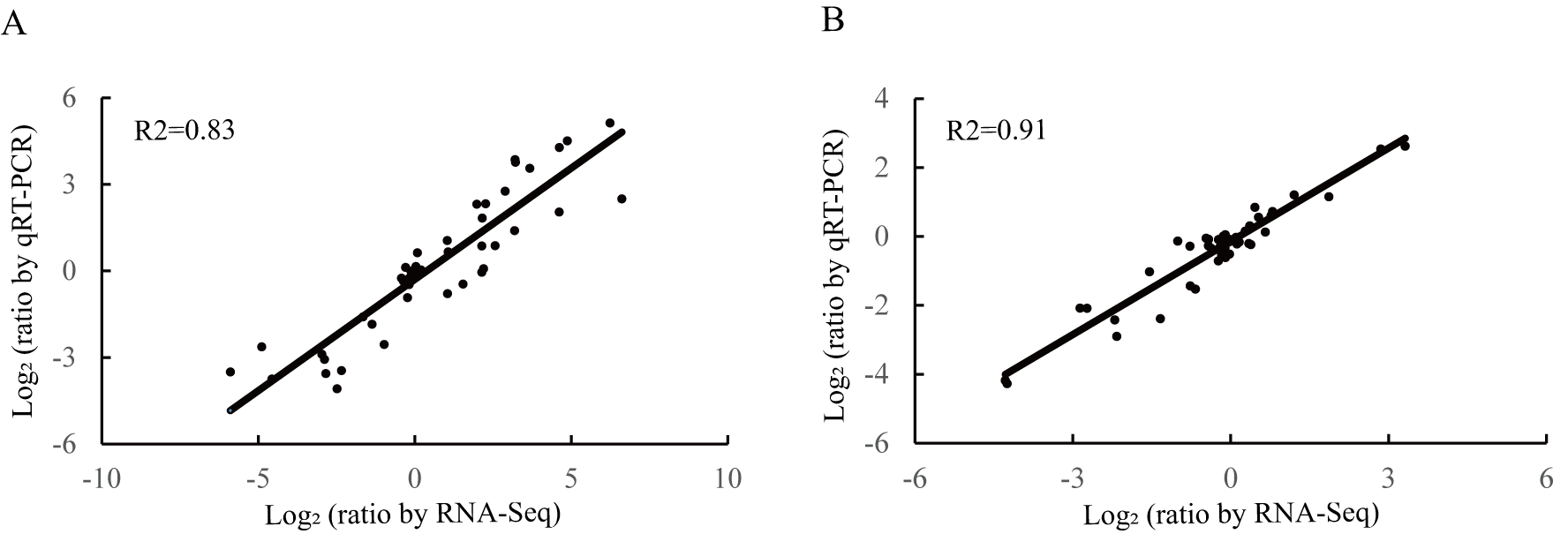

Supplement: FIGURE S4 — Verification of the transcriptome results through the experiments of qRT-PCR. The relationships between qRT-PCR and RNA-seq. Values were the log2 ratio (FPKM ratio by RNA-Seq) for genes in rice (A) and Arabidopsis (B). The determine coefficient (R2) was indicated in the figure. All qRT-PCR reactions were performed in three biological replicates. [file Image_4.TIF]
